# A gain series method for accurate EMCCD calibration

**DOI:** 10.1038/s41598-021-97759-6

**Published:** 2021-09-15

**Authors:** Duncan P. Ryan, Megan K. Dunlap, Martin P. Gelfand, James H. Werner, Alan K. Van Orden, Peter M. Goodwin

**Affiliations:** 1 Center for Integrated Nanotechnologies, Los Alamos National Laboratory, Los Alamos, 87545 USA; 2 Department of Chemistry, Colorado State University, Fort Collins, CO 80523 USA; 3 Department of Physics, Colorado State University, Fort Collins, CO 80523 USA

**Keywords:** Imaging and sensing, Microscopy, Single-molecule biophysics

## Abstract

Calibration of the gain and digital conversion factor of an EMCCD is necessary for accurate photon counting. We present a new method to quickly calibrate multiple gain settings of an EMCCD camera. Acquiring gain-series calibration data and analyzing the resulting images with the EMCCD noise model more accurately estimates the gain response of the camera. Furthermore, we develop a method to compare the results from different calibration approaches. Gain-series calibration outperforms all other methods in this self-consistency test.

## Introduction

The electron-multiplying charge-coupled device (EMCCD) provides sensitive photon detection in low-light conditions^1^. In scientific applications, EMCCDs are more adaptable than conventional CCDs because they amplify signals from detected photons, producing higher signals from photons than other noise sources. The amplification level can be adjusted and optimized for the imaging conditions. In microscopy applications, EMCCD cameras can detect emission from single fluorophores where emission may be weak. These devices have enabled numerous imaging modalities, and have helped enhance the spatial resolving power of microscopy methods in the biological and material sciences^2–7^. EMCCDs have also found use in quantum imaging and sensing where spatially-resolved single-photon detection^8^ is used in pair-correlation experiments^9^. The defining feature of the EMCCD—the amplification of small numbers of photoelectrons above electronic noise—maintains the linear relationship between signal level and the incident photon flux. Accurate characterization of the amplification process and the conversion to digital units are necessary for comparing intensity levels among pixels^10,11^, such as when fitting point-spread functions for super-resolution localization microscopy^2^ or when comparing intensity differences across regions of the sensor for quantum correlation detection^12,13^.

The detection of photons and the amplification of the resulting photoelectrons are both stochastic processes contributing to fluctuations of a signal (noise). Most calibration methods leverage the mathematical relationship of the mean of the signal to its variance, derived from the complete EMCCD noise model^2,8^, to determine the analogue-to-digital unit (ADU) conversion factor and the gain of the amplification register. These characterization methods are referred to as mean-variance (MV) tests and work by applying the photon transfer curve (PTC) to a series of datasets of varying intensity levels^14–18^. Manufacturers and researchers typically make use of these calibration methods because they are easy to implement^19–21^.

Although widely used, MV tests have limitations and have been identified as problematic for calibration^22^. MV methods are based on linear regressions. As such, parameter estimation and uncertainty analysis reflect the implied noise of measuring the means and variances from each dataset, and indirectly reflect correlations among parameters that stem from the EMCCD noise model. Covariances among parameters are not accurately addressed using MV methods, potentially leading to the mischaracterization of parameters and their uncertainties^23–25^. Alternative calibration strategies or imposing consistency requirements are also difficult within the MV framework. Furthermore, as a matter of calibration expediency, MV tests require many measurements per gain set-point that are time-consuming to obtain, and researchers will typically make use of several gain settings depending on experimental conditions to obtain optimal signal levels. This can be particularly inconvenient because voltage degradation in the amplification register requires periodic recalibration^26^. A method that characterizes multiple camera parameters in fewer measurements and with improved accuracy is therefore highly desirable. Finally, it is important to have an independent test of parameter estimates to provide confidence in the results generated by a given calibration method.

We introduce a probabilistic calibration method for determining the ADU and gain parameters of a camera using the EMCCD noise model. Comparing various calibration approaches, we found that parameter estimates derived from the same dataset can vary significantly depending on the chosen calibration method. We demonstrate that a new gain-series calibration outperforms other methods, based on independent consistency measurements from localization experiments. This method is also more expedient for calibrating multiple gain set-points than intensity-series based approaches, such as MV tests.

## Theory

The architecture of EMCCD cameras has three primary stages: the accumulation of photoelectrons (electrons generated directly from photons) in each pixel of the detector, the movement of those charges through an amplification register that multiplies the number of electrons, and the readout of the post-amplification charges as a digital count. See Supplementary Section S1 for a reference of EMCCD device architecture. Each stage introduces noise unique to its operation. While several sources of noise can be present in addition to the photo-generated charges, such as clock-induced-charge (CIC or spurious charge) and thermal noise (dark noise), their impacts on the output signal are still categorized by these stages of EMCCD operation and can be included in more comprehensive formulations of the noise model.

The combined processes of emission and detection produce photoelectrons in the detector pixels with a Poisson distribution (shot noise). Amplification is generally modeled using a gamma distribution (for a more comprehensive discussion about the appropriate form, see Hirsch et al.^27^). The distribution of the number of electrons after these two stages has the form of a Poisson-gamma (PG) distribution^28,29^ and is often used alone as an approximation of the complete noise model when the PG noise is much larger than the readout noise. Readout noise is normally-distributed and the resulting signal is the convolution of the PG distribution with a normal distribution; thus the complete noise model of an EMCCD camera is a Poisson-gamma-normal (PGN) distribution^27^. When amplification is disabled, the complete noise model is reduced to a Poisson-normal (PN) distribution.

In an EMCCD, charges are moved through individual wells (sub-units of a register) serially and, aside from the initial pixel separation in the sensor array, all charges are moved through common wells and registers that define the camera response. In theory, the readout of each pixel should differ by only the photoelectron generation rate, with the amplification and readout registers responses invariant of the source pixel. The effects of well capacity (saturation), in either the individual pixels or the amplification register, are not represented in the PGN model.

### Camera noise model

In scaled units, the PGN noise model is^27,28^

1 $$\begin{aligned} p(S|E, g, \phi , S_0, \sigma )&=q(S|E, g, \phi )*N(S|S_0, \sigma ), \end{aligned}$$

where * is the convolution operator, and *q*(*S*) is either the Poisson-gamma distribution

2a $$\begin{aligned} q(S|E, g, \phi )&=\phi \,P_0(E)\,\delta (S\phi )+\phi \sum _{n=1}^{\infty }P_n(E)\,\Gamma _n(S|g, \phi ) \end{aligned}$$

2b $$\begin{aligned} = \phi \exp \left( { - E} \right){\mkern 1mu} \delta (S\phi ) + \left( {\frac{{E\phi }}{{gS}}} \right)^{{\frac{1}{2}}} \exp \left( { - \frac{{S\phi }}{g} - E} \right){\mkern 1mu} I_{1} (2\sqrt {ES\phi /g} ) \end{aligned}$$

for the total number of electrons after the multiplying register (PGN noise model) or the Poisson distribution

2c $$\begin{aligned} q(S|E, 1, \phi )&=\phi \dfrac{E^{S\phi }\exp {\left( -E\right) }}{(S\phi )!} \end{aligned}$$

for the total number of photoelectrons in non-multiplying measurements (PN noise model). *N*(*S*) is the normally distributed readout noise

3 $$\begin{aligned} N(S|S_0, \sigma )&=\dfrac{1}{\sqrt{2\pi }\sigma }\exp \left( -\dfrac{(S-S_0)^2}{2\sigma ^2}\right) \end{aligned}$$

where the constant offset $S_0$ is incorporated into the readout noise term. All variables used above are defined in Table 1. Only *S* is restricted to non-negative integers while the remainder of the variables are positive real numbers. In these forms, the raw data in digital count units is used in the noise model without additional conversion (e.g. offset subtraction or conversion into units of electron numbers). The additional ADU conversion factors $\phi$ ensure normalization of the scaling transformation from ADU to electron numbers. Equation (1) was used to evaluate likelihood values for the probabilistic calibration methods below. No analytical solution exists for Eq. (1). To use an approximation-free noise model for the evaluation of likelihoods, in this work we perform the computational convolution using fast Fourier transforms (FFTs). Using units of counts instead of electron numbers means that no discretization artifacts arise and the application of a discrete FFT is appropriate. Additional implementation notes are presented in the Supplemental Information Section S2.

**Table 1:** Variable definitions.

*S*	Pixel value [counts]
$S_0$	Offset [counts]
*E*	Photoelectrons
*g*	Gain
$\phi$	ADU conversion factor [photoelectrons/count]
$\sigma$	Standard deviation of readout noise [counts]

We introduce probabilistic calibration methods that use maximum likelihood estimation (MLE) to group multiple intensity datasets (for ADU or gain calibration) or multiple gain datasets (for gain calibration only) together with shared parameters using a global log-likelihood function

4 $$\begin{aligned} \ln \mathcal {L}^\text {global}=\sum _{i=1}^{M_\text {max}}\;\sum _{j=1}^{N_\text {max}}\ln \mathcal {L}(S^{(i)}_j|\mathbf {p}^{(i)}), \end{aligned}$$

where $M_\text {max}$ is the number of individual datasets in the series, $N_\text {max}$ is the number of samples (frames) in an individual series, and $\mathbf {p}^{(i)}$ represents the combined set of common parameters and unique parameters for a given dataset in a series. Optimization was used to find parameter estimates that maximized the global log-likelihood function.

Gain calibration using the EMCCD noise model Eq. (1) has previously^2,30^ been used to fit single intensity level calibration data by merging pixels into a combined empirical distribution function—inherently assuming spatially homogeneous illumination over a subset of the pixels. However, we found no specific reference to MLE-based calibration using intensity-series methods.

**Figure 1:**
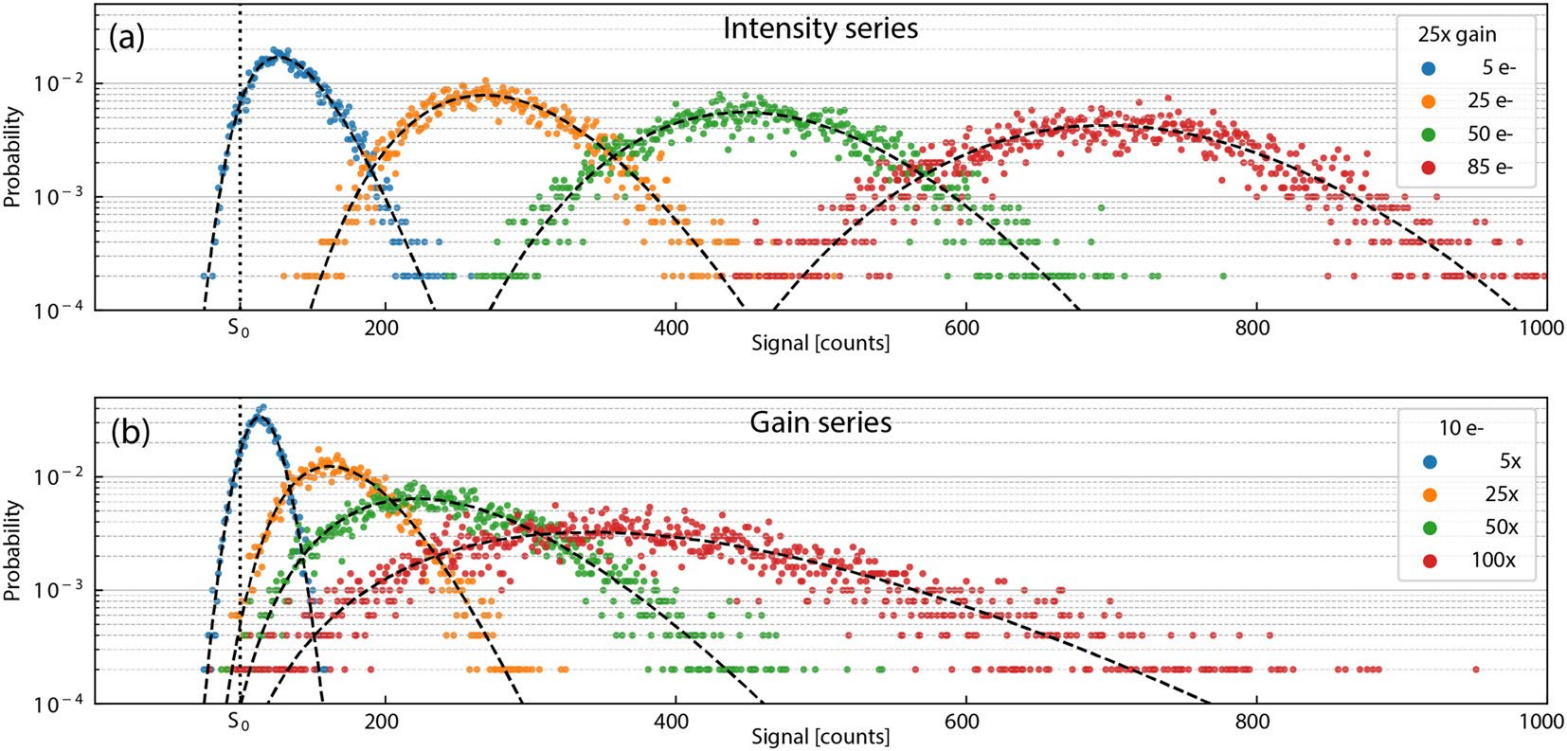
Series concept for parameter estimation. (**a**) An IS calibration collects samples from multiple intensity levels sharing a common gain value. Histograms of simulated samples at 25× gain (colored markers) and their corresponding PDFs (dashed lines) for four intensity levels are depicted. For larger photoelectron values, the Poisson distribution of the PGN model more closely resembles a normal distribution and the skewness, always positive for in the PGN model, decreases while the variance increases. (**b**) A GS calibration applies the same concept of grouping multiple measurements, but assumes independent gain parameters sharing a common photoelectron value. Depicted are the simulated histograms and PDFs for four gain levels representing a common intensity of 10 photoelectrons. For a constant intensity level, as gain increases, the skewness and variance both increase. The IS and GS depicted were simulations of 5000 samples, with $\sigma ={10}{counts}$, $S_0={100}{counts}$, and $\text {ADU}={3.5}{e^- / count}$, representing similar parameter spaces and sample sizes as the cameras experimentally tested.

In this work, we report the pixel-wise application of multi-dataset MLE methods (for ADU and gain calibration) and additionally introduce a gain-series approach that is entirely new. An intensity-series (IS) calibration uses measurements at a fixed gain value and different illumination intensity levels to change the statistical distribution of the output signal *S*. This approach forces a shared gain value among all datasets. A gain-series (GS) calibration, in contrast, uses different gain set-points to change the statistical distribution of the output signal *S* while maintaining a fixed illumination intensity on the camera. Figure 1 illustrates how the distribution functions, Eq. (1), change shape for the intensity-series methods and the gain-series method. MLE is sensitive to such higher-order moments (distribution shapes) whereas MV tests are not. Additionally, we explored a hybrid GS approach that partially relaxes the shared illumination intensity assumption to accommodate potential illumination drift between datasets. Details about this hierarchical GS approach are described in the Supplementary Information Section S2.

The model parameters for each probabilistic calibration method are summarized in Table 2. To isolate analysis of the individual stages in an EMCCD, readout noise was separately characterized from dark images. The mean and variance of an individual pixel over a series of dark frames determine $S_0$ and $\sigma$, respectively. Thus, MLE optimization used *E*, *g*, and $\phi$ as free variables, fixing $S_0$ and $\sigma$ to values determined from dark images (see Fig. S3).

**Table 2:** Parameter sets for MLE. Shared and unique parameters for the multiple dataset likelihood-based calculations. IS and GS MLE methods use different combinations of shared parameters to group datasets.

$\mathbf {p}^{(i)}=\{E^{(i)}, g=1, \phi , S_0, \sigma \}$	IS MLE for $\phi$ calibration
$\mathbf {p}^{(i)}=\{E^{(i)}, g, \phi , S_0, \sigma \}$	IS MLE for gain calibration
$\mathbf {p}^{(i)}=\{E, g^{(i)}, \phi , S^{(i)}_0, \sigma ^{(i)}\}$	GS MLE for gain calibration
$\mathbf {p}^{(i)}=\{E^{(i)}\sim \mathcal {N}(\overline{E}, \sigma _{\overline{E}}), g^{(i)}, \phi , S^{(i)}_0, \sigma ^{(i)}\}$	hierarchical GS for gain calibration

### Mean–variance relationships

For comparison with traditional calibration methods, we calculated parameters using MV tests. MV methods use expressions for the mean of a distribution and its variance that can be analytically derived. For the ADU conversion factor, these expressions can be found using the moment generating function of the Poisson distribution and Eq. (2c), yielding the relationship

5a $$\begin{aligned} \sigma ^2_{S, \phi }=\dfrac{\langle S\rangle }{\phi }. \end{aligned}$$

Similarly, for gain calibration the moment generating functions of the gamma distribution and Eq. (2a) yield the relationship

5b $$\begin{aligned} \sigma ^2_{S, g}=\dfrac{2g\langle S\rangle }{\phi }. \end{aligned}$$

**Figure 2:**
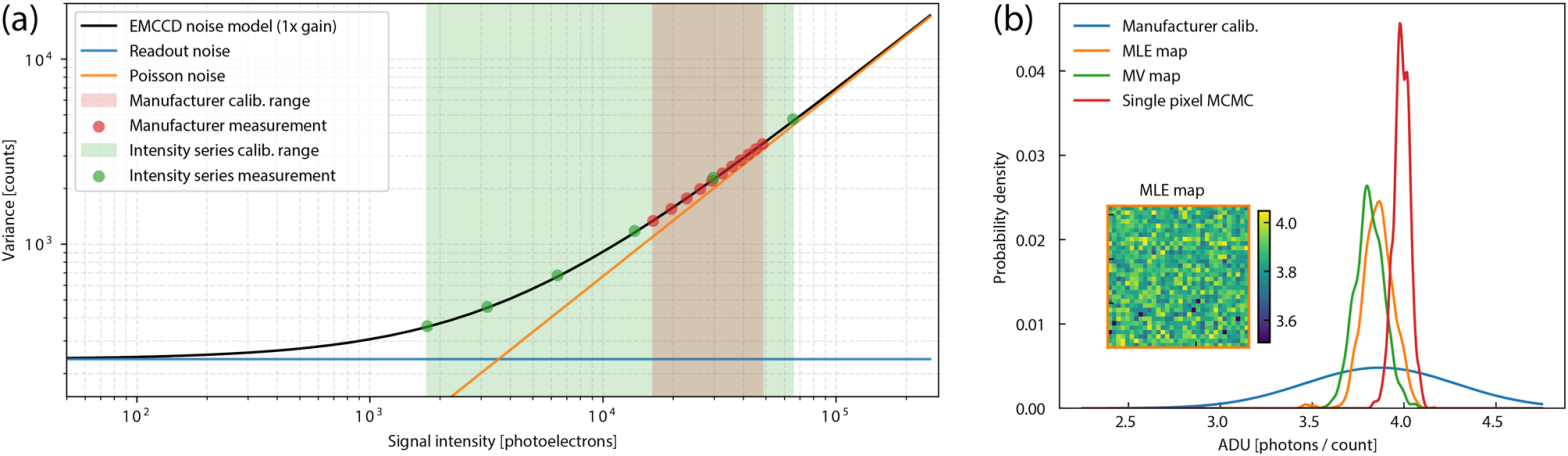
ADU calibration. (**a**) The photon transfer function for ADU calibration. Readout noise (blue) is independent of signal intensity while Poisson noise (orange) is linearly proportional to the signal intensity. The manufacturer calibration range encompassed a small range of the signal intensity and was linearly spaced, which weighs the data from high signal intensities more heavily. Calibration measurements for this work (green) spanned a broader range and were logarithmically distributed. (**b**) Distributions of ADU estimates determined using the MLE method on each pixel (orange), the MV test on each pixel (green), and the manufacturer calibration data (blue). MCMC inference on a single pixel (red), shows the uncertainty associated with an individual measurement. The inset MLE map emphasizes the calibrated ADU values are uniform across the sensor area despite pixel-wise variations in the photon flux (image patterns due to the source).

MV tests use the IS approach, thereby measuring multiple noise levels, and fit the linear expression

6 $$\begin{aligned} \sigma ^2_\text {total}=\sigma ^2_\text {readout}+\sigma ^2_{S,k} \end{aligned}$$

to the calculated means and variances from each dataset, where *k* indicates the parameter being calibrated ($\phi$ or *g*). The slope of the linear fit is proportional to the parameter of interest through Eqs. (5a) or (5b). Figure 2a illustrates this concept for ADU calibration. With the absence of a specific sampling noise model, this linear regression procedure implicitly assumes normally-distributed sample noise (of the mean and variance values), which differentiates MV methods from the probabilistic approach where all noise is described by the camera noise model.

## Results

We applied MV methods and MLE methods to calibrate a Princeton Instruments ProEM 512B+ EMCCD camera (2012 manufacture date). For comparison, a second camera, a ProEM 512B (2009 manufacture date), was additionally calibrated and tested. Results for the second camera are available in the Supplementary Information Section S11.

### Analogue-to-digital unit (ADU) conversion factor

The ADU conversion factor $\phi$ must be determined before camera gain can be calibrated. Intensity-series datasets were acquired and analyzed pixel-wise using (1) the traditional MV method and (2) the MLE method described above. Details are described in the Methods section. ADU calibration used the MLE parameter set $\mathbf {p}^{(i)}=\{E^{(i)}, g=1, \phi , S_0, \sigma \}$. That is, the PN noise model where $E^{(i)}$ is the photon intensity of the *i*-th intensity level of the series, and all intensity datasets share the same ADU value $\phi$, offset $S_0$, and readout noise $\sigma$.

Figure 2b shows the ADU calibration results using both methods on the same dataset, as well as the manufacturer calibration data. The MV and MLE plots show distributions of the ADU parameter $\phi$ generated by combining calibration results from all pixels in the 32 $\times$ 32 array (see insert map and Supplementary Information Section S5). Thus, the $\phi$ parameter uncertainty is depicted by effectively running 1024 experiments. For this camera, the MV and MLE calibrations generated similar results and were consistent with the manufacturer calibration. Both MV and MLE methods provided more precise estimates of the ADU value than the manufacturer. The other camera we tested did not show such agreement and the readout architecture of the camera produced unexpected results (see Fig. S10).

Markov chain Monte Carlo (MCMC) inference can indicate parameter uncertainties inherit to the single-pixel data by sampling the likelihood function in a random walk process. MCMC results from a single pixel, the red curve in Fig. 2b, illustrate the ADU uncertainty intrinsic to the camera noise model and the calibration dataset. Increasing the number of samples and the number of illumination levels can improve the parameter uncertainty up to a point, however the EMCCD noise model dictates the degree of coupling between parameters. Covariance among $\phi$ and the individual intensity levels $E^{(i)}$ ultimately limit the precision of the ADU conversion factor.

### Gain calibration

The electron-multiplying gain factor *g* was calibrated using MV and MLE methods. MLE for the IS dataset used the parameter set $\mathbf {p}^{(i)}=\{E^{(i)}, g, \phi , S_0, \sigma \}$; that is, only the photoelectron generation rates $E^{(i)}$ were unique to each intensity level and all other parameters were shared. The parameter set for MLE on the GS dataset was $\mathbf {p}^{(i)}=\{E, g^{(i)}, \phi , S_0^{(i)}, \sigma ^{(i)}\}$. Because of the observed correlation between the gain set-point and the offset $S_0$ (see Supplementary Information Section S4), the parameters $S_0^{(i)}$ and $\sigma ^{(i)}$ were determined for each gain set-point; although, $\sigma ^{(i)}$ was largely insensitive to the gain. GS analyses that included a 1$\times$ dataset and that excluded this dataset were both tested. The former forces an additional level of self-consistency with the Poisson-normal (PN) noise model, while the latter was tested in case camera readout was unexpectedly different when the gain was turned-off but charges still moved through the multiplication register.

A non-centered hierarchical (NCH) calibration method using the same GS dataset was also explored to relax the shared intensity requirement by using $E^{(i)}\sim \mathcal {N}(\overline{E}, \sigma _{\overline{E}})$, where optimization was over the hyperparameters $\overline{E}$ and $\sigma _{\overline{E}}$ instead of the individual intensities $E^{(i)}$ (a maximum *a posteriori* estimation). The NCH method was tested because GS measurements require the illumination source to be steady for longer durations than IS methods while each gain set-point is acquired. While no evidence of signal drift was observed in the measurements, the NCH method was tested for comprehensiveness. Figure S5 shows statistical stationarity tests demonstrating the steadiness of the illumination source used.

The calibration-on-the-spot (COTS) method by Mortensen and Flyvbjerg^31^ was additionally used to calibrate camera gain. This approach allows gain parameters to be estimated without separate calibration datasets and is useful when analyzing single-particle images where the calibration is not known. COTS was performed directly on the bead data described later for validation measurements.

**Figure 3:**
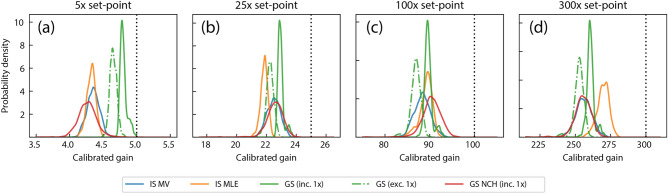
Results from gain calibration methods. Distributions of the gain parameters for (**a**) 5×, (**b**) 25×, (**c**) 100×, and (**d**) 300× set-points are shown for the MV test (blue), intensity series MLE (orange), gain series MLE (green), and MAP of the gain series using a hierarchical model (red). Each method produced differing estimates of the gain parameters. Estimates from each method were significantly smaller than the software set-points (dotted black).

Figure 3 shows the calibration results for five gain set-points (5×, 25×, 100×, and 300×) using intensity-series based MV and MLE methods, and gain-series based MLE methods. Distributions were generated from the 1024 individually analyzed pixels, demonstrating the uncertainties associated with the parameter estimates. Single-pixel MCMC inference showed similar levels of uncertainty relative to the collective distributions as ADU calibrations, and are not displayed for brevity. Each calibration method recovered different estimates of the effective gains. Note that the two IS calibration methods used the same datasets, and all GS calibration methods used a different, but common dataset. A second camera tested showed greater dissimilarities among the methods (see Fig. S11). The COTS calibration method generated gain estimates that were significantly higher than the dedicated calibration methods and are not shown in Fig. 3 for visual scaling convenience. Figure S9 shows the calibration results in full.

## Discussion

Given the dissimilar gain estimates generated by each method, some additional criteria must be used to select the optimal parameter set. Indeed, the gain parameter distributions, Fig. 3, are narrowly distributed and show little overlap among some of the methods. We introduce a new localization-based validation method to provide an independent self-consistency test for gain calibration method selection.

The point-spread function (PSF) produced by an individual emitter is composed of spatially varying intensity levels. Localization analysis optimizes the parameters of a particular PSF image model to determine the best match between measured signal intensities in a spot and their expected intensities given by the PSF. This is, in fact, similar in concept to the IS calibration methods described above: matching a data series (the individual pixels of the emission spots) to different intensity levels; although, the intensity levels are distributed according to the PSF. A single, overall intensity parameter scales the PSF to match the signal levels. PSF fitting using likelihood estimation (as opposed to non-probabilistic methods, e.g. least-squares fitting) can use the EMCCD noise model, Eq. (1), to estimate the probability of a pixel value given the parameters of the camera (*g*, $\phi$, $S_0$, and $\sigma$). Thus, a likelihood estimator will produce the most accurate fit when the parameters are correctly specified.

Gain-series datasets of isolated emitters at constant fluorescence intensity yield a self-consistency test for the calibration results. If the gain estimates are valid, intensities recovered from PSF fitting would most accurately reflect the photon flux in the images, and the fluxes are independent of the gain set-point. This approach is a validation of the calibration methods independent of the calibration datasets themselves, and is relevant to many applications of EMCCDs. Thus, the hybrid IS/GS requirements of this method are not inherently biased toward one calibration scheme over another.

**Figure 4:**
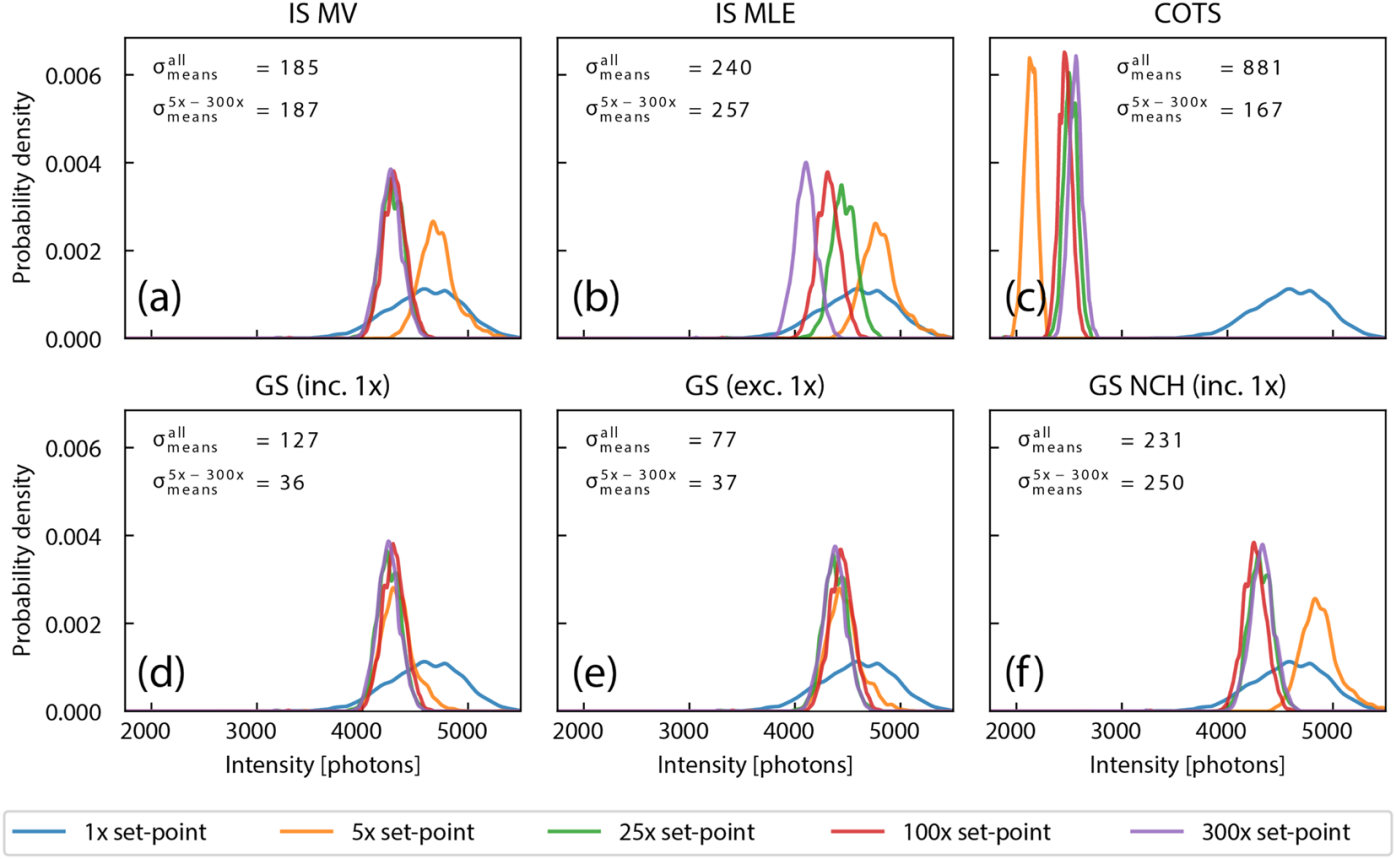
Consistency results from bead localization. Intensity estimations from fitting a single fluorescent bead imaged at a fixed excitation intensity at different gain set-points. Distributions are composed of fitting the same bead over multiple frames. For an ideal calibration, the distributions should overlap completely, regardless of the gain set-point. Only the gain-series methods demonstrate this consistency. The standard deviations of the distribution means, $\sigma _\text {means}$, are indicated for each method (including and excluding the 1× distribution) as a metric for the performance of the various methods.

Figure 4 shows the intensity distributions of a representative single fluorescent bead imaged at each gain set-point using the calibrated camera parameters from the methods described previously. Each parameter set was tested against the same image data, and COTS calibrations were determined directly from the image data of the individual bead. The best overlap of all intensity levels occurred for the GS methods; although the NCH method appears to be overly permissive and the 5× results were outliers similar to the IS methods. The standard deviation of the means are indicated in each plot to quantitatively depict the effectiveness of each calibration method.

**Figure 5:**
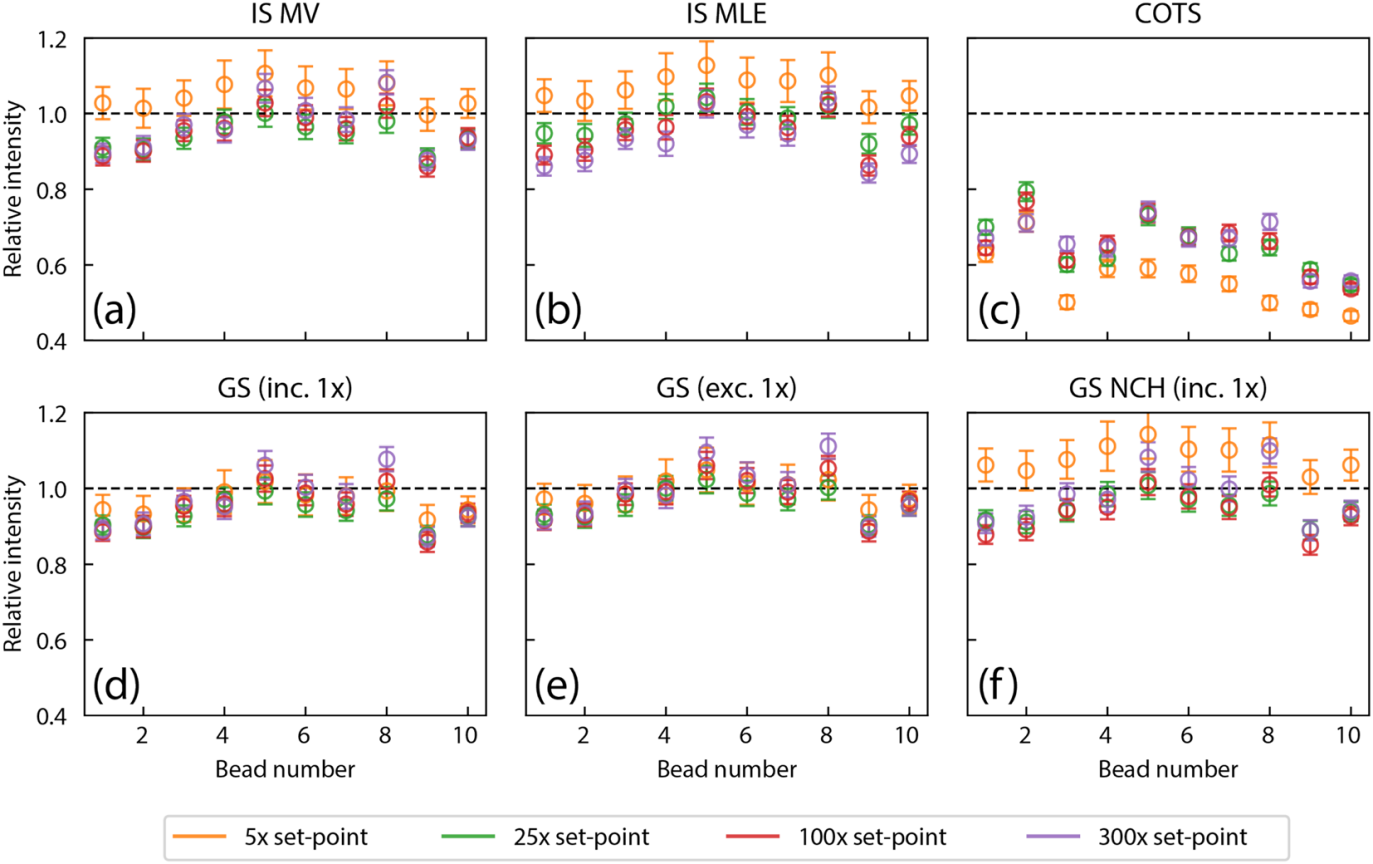
Summary of consistency results for a collection of beads. Plots show the results of localization fitting for ten beads imaged together in the same field of view, scaled to the mean intensity from the 1× measurements. Intensity values from the 5× dataset are outliers for all calibration methods except the two gain-series. The gain-series methods show consistency across gain set-points for all beads examined.

Figure 5 summarizes the intensity distributions for ten beads measured in the same dataset, scaled to the mean intensity of the 1× distribution. For the ensemble of beads, the GS methods performed better with smaller spreads (with the exception of the NCH method). The 5× measurements were outliers for all but the GS methods. Although the GS methods that included and excluded a 1× dataset performed similarly on this camera, there were clearer differences between the two methods for the second camera calibrated, indicating the inclusion of a 1× dataset improves results (see Fig. S12).

The 5× results were consistent outliers in the IS methods, the COTS method, and the NCH GS method. This discrepancy may be explained by examining the noise model in detail. While the PGN model in Eq. (1) is the widely accepted model for EMCCD noise, it is actually an approximation of a Bernoulli process that models the impact ionization for charge amplification. The amplification process is responsible for the “excess noise” factor of EMCCDs: i.e. the factor of 2 in Eq. (5b). However, at low gain set-points, a Bernoulli treatment of the amplification process shows that the excess noise factor is not constant-valued^32,33^. Indeed, the excess noise factor deviates significantly from the value of 2 in 5× measurements (see Supplementary Information Section S3), rendering the PGN noise model an inaccurate representation in such operating conditions. Because GS methods estimate the signal strength through the combination of several set-points, particularly those where the noise model is more accurate, this deviation is likely lessened for the GS methods compared to the IS methods. All of the calibration methods considered in this work are fundamentally based on the same noise model, and the different gain estimates of each method may ultimately stem from the limitations of this model to perfectly describe the behavior of an EMCCD.

The COTS calibration method is an ingenious solution for uncalibrated datasets, but did not perform as well as the IS or GS methods explored in this work. The PSFs in our bead measurements were spread across fewer pixel than the COTS technique was originally demonstrated on, producing fewer sample variances that can be used to estimate parameters. A different imaging configuration might have given more favorable results for COTS.

## Conclusions

Proper EMCCD calibration is necessary in many microscopy and imaging applications. For example, when images are simultaneously recorded by multiple cameras, such as in multi-color imaging^34,35^ or in spectral deconvolution imaging^36^, the relative intensities between different camera images are critical, and raw signals must be scaled by the true amplification values for quantitative interpretation. The correct scaling involves the calibration of the ADU factor and the effective gain multiplier value.

We found that parameter estimates derived from probabilistic models of GS calibration data performed better than MV tests, IS MLE, and COTS methods in localization-based intensity consistency checks. Distributions of bead intensity estimations using GS calibration values more closely overlapped with one another, and the inclusion of a 1× dataset for GS calibration produced the strongest consistency in a second camera. GS methods use fewer datasets than other calibration methods, requiring only a single measurement from each gain set-point of interest, increasing the speed and ease of the calibration process.

The calibration methods used in this work are included as Code 1 and can be found online at https://github.com/ryand-LANL/EMCCD-calibration.

## Methods

Datasets for ADU and gain calibration were acquired by exposing EMCCD cameras to a range of illumination levels or acquiring image series at different gain set-points with the same illumination level. The sensors were illuminated in a trans-illumination microscope configuration for calibration, imaging a diffuse LED source through an oil-immersion objective (Olympus Apo N 60×, 1.49 NA). Image series were acquired over a 32 × 32 pixel region in the center of an EMCCD sensor with a flat-top illumination profile. In this way, edge-effects from the hardware-cropped readout region were more obvious, and the calibration methods tested against such artifacts. For example, we observed a dependence of the offset parameter $S_0$ on row-position (slightly lower offset for the initial pixels in a row), but the analysis showed no indication of the behavior affecting calibration results (i.e. the results were spatially homogeneous). See Supplementary Information Section S5 for additional details. Such a limited readout region also reduced analysis time and data storage while providing more than a sufficient number of pixels to characterize statistical properties.

The initial 1000 frames were discarded from each acquisition to allow the cameras to stabilize at operating voltages and clock timings, and all calibration procedures were based on 5000 frames of data. The EMCCD sensors were illuminated for 40 ms of a 50 ms exposure using a triggered 590 nm LED source to avoid additional illumination during readout (pixel bleeding). The cameras were operated in frame-transfer readout mode to further avoid bleeding.

Typically, exposure time is adjusted to control the number of generated photoelectrons. Because this procedure alters the internal timing of a camera, potentially affecting the camera response, we elected to vary the illumination intensity directly. Illumination intensities were logarithmically spaced to emphasize the PDF shapes that are more substantial at lower intensities. Changes to the camera gain set-points and the LED intensity were allowed to settle for several minutes, with the cameras acquiring images, before recording data. The LED source produced steady illumination and no evidence of intensity drift was observed over the duration of individual measurements. Cameras were recalibrated according to the manufacturer’s protocols immediately before calibration datasets were measured.

### Intensity-series measurements for calibration

ADU calibration datasets were acquired at a 1× gain set-point where readout noise and Poisson noise are the only sources of signal noise. IS gain calibration datasets were taken for each gain set-point. Six intensity levels were used for ADU and IS gain calibration. The readout offset parameter $S_0$ and the readout noise $\sigma$ were determined from a second measurement by shuttering the camera and recording the mean and variance without illumination. The readout noise parameters were fixed to these values for both MV and MLE calibration methods. While these parameters should be the same for all pixels, in practice the readout order affected $S_0$, particularly for pixels near the edges of the readout area. These features appeared regardless of the image cropping during acquisition or in post processing. Therefore, each pixel was calibrated independently using their unique $S_0$ and $\sigma$ values.

### Gain-series measurements for calibration

Five gain set-points were taken for a complete GS calibration series: 1×, 5×, 25×, 100×, and 300×. The LED illumination source was held constant and allowed to stabilize before measurements were taken. The illumination intensity was set below saturation for the highest gain set-point to avoid full-well artifacts (aiming for $\sim 2^{15}$ counts on the 16-bit dynamic range). Dark images with the shutter closed were recorded immediately after each gain set-point for the $S_0$ and $\sigma$ image maps. The mean ADU values from the MLE analysis method was used to analyze GS datasets.

### Bead measurements

0.5 ${\upmu }{m}$ fluorescent beads (Fluoresbrite YG Microspheres, Polysciences) were dispersed onto glass coverslips and excited with a 488 nm CW laser (Coherent) in an epi-illumination configuration. The magnification of the microscope was 70×. 500 frames were recorded for each gain set-point and there was no evidence of intensity fluctuations over the duration of the gain-series or during a single gain measurement. A 50/50 beamsplitter was used in the detection path to image the beads on the two calibrated cameras for simultaneous measurements in a dual-view configuration. Thus, the same beads at the same times are compared between Figs. 5 and S12.

Bead intensities were determined in each frame using a custom super-resolution fitting algorithm that implemented the complete PGN noise model without approximation for the readout convolution. Spots were fit in a first-pass to a pixel-integrated Gaussian PSF. The spot images were then subtracted from the estimated PSFs and the differences stored as over-sampled residual images. A second-pass fit where the pixel-integrated Gaussian PSF was augmented with the residual image was then performed and intensities extracted from the fit. The residual compensation ensured that the number of photons was not under- or over-estimated due of the PSF model. However, because PSF model mismatch is relative, the same results were obtained with and without the residual compensation. While the beads used in this work were sub-diffraction limited, larger emitters could be used, so long as the spots generated exhibit intensity variation. Thus, beads larger than the diffraction limit could also be used, as the spatial profile of their emission is a convolution of a spherical object and the optical transfer function of the imaging system.

## Supplementary Information

Supplementary Information.

## References

[1] Denvir, D. J. & Conroy, E. Electron-multiplying CCD: The new ICCD. in *Low-Light-Level and Real-Time Imaging Systems, Components, and Applications*, vol. 4796, 164–174, (International Society for Optics and Photonics, 2003). 10.1117/12.457779.

[2] Ulbrich MH, Isacoff EY (2007). Subunit counting in membrane-bound proteins. Nat. Methods.

[3] Han, J. J., Shreve, A. P. & Werner, J. H. Super-resolution optical microscopy. in *Characterization of Materials*, 1–15 (American Cancer Society, 2012).

[4] Chao J, Sally Ward E, Ober RJ (2016). Fisher information theory for parameter estimation in single molecule microscopy: Tutorial. J. Opt. Soc. Am. A.

[5] Lee A, Tsekouras K, Calderon C, Bustamante C, Pressé S (2017). Unraveling the thousand word picture: An introduction to super-resolution data analysis. Chem. Rev..

[6] Ryan DP (2018). Mapping emission from clusters of CdSe/ZnS nanoparticles. J. Phys. Chem. C.

[7] Sage D (2019). Super-resolution fight club: Assessment of 2D and 3D single-molecule localization microscopy software. Nat. Methods.

[8] Plakhotnik T, Chennu A, Zvyagin AV (2006). Statistics of single-electron signals in electron-multiplying charge-coupled devices. IEEE Trans. Electron Dev..

[9] Avella A, Ruo-Berchera I, Degiovanni IP, Brida G, Genovese M (2016). Absolute calibration of an EMCCD camera by quantum correlation, linking photon counting to the analog regime. Opt. Lett..

[10] Mullikin, J. C. *et al.* Methods for CCD camera characterization. in *Image Acquisition and Scientific Imaging Systems*, vol. 2173, 73–84. (International Society for Optics and Photonics, 1994)10.1117/12.175165.

[11] Heintzmann, R., Relich, P. K., Nieuwenhuizen, R. P. J., Lidke, K. A. & Rieger, B. Calibrating photon counts from a single image. arXiv:1611.05654 [astro-ph, physics:physics] (2016).

[12] Meda A (2017). Photon-number correlation for quantum enhanced imaging and sensing. J. Opt..

[13] Kumar A, Nunley H, Marino AM (2017). Observation of spatial quantum correlations in the macroscopic regime. Phys. Rev. A.

[14] Reibel Y, Jung M, Bouhifd M, Cunin B, Draman C (2003). CCD or CMOS camera noise characterisation. Eur. Phys. J..

[15] Janesick, J. R. *Photon Transfer: DN* → λ, 2nd edn. (SPIE Press, 2010).

[16] DeWeert, M. J., Cole, J. B., Sparks, A. W. & Acker, A. Photon transfer methods and results for electron multiplication CCDs. in *Applications of Digital Image Processing XXVII*, vol. 5558, 248–259 (International Society for Optics and Photonics, 2004). 10.1117/12.562223.

[17] Huang Z-L (2011). Localization-based super-resolution microscopy with an sCMOS camera. Opt. Express.

[18] Li L, Li M, Zhang Z, Huang Z-L (2016). Assessing low-light cameras with photon transfer curve method. J. Innov. Opt. Health Sci..

[19] Long F, Zeng S, Huang Z-L (2012). Localization-based super-resolution microscopy with an sCMOS camera. Part II: Experimental methodology for comparing sCMOS with EMCCD cameras. Opt. Express.

[20] Mehta SB (2016). Dissection of molecular assembly dynamics by tracking orientation and position of single molecules in live cells. PNAS.

[21] Liu S (2017). sCMOS noise-correction algorithm for microscopy images. Nat. Methods.

[22] Watanabe, S., Takahashi, T. & Bennett, K. *Why Camera Calibration Using Variance Doesn’t Work for Photoresponse Calibration of Scientific CMOS (sCMOS) Cameras (and Other Photodetectors)*. http://www.hamamatsu.com/sp/sys/en/documents/FOM2017_Poster.pdf (2017).

[23] Long F, Zeng S, Huang Z (2014). Effects of fixed pattern noise on single molecule localization microscopy. Phys. Chem. Chem. Phys..

[24] Basden, A. Analysis of EMCCD and sCMOS readout noise models for Shack-Hartmann wavefront sensor accuracy. arXiv:1506.07929 [astro-ph] (2015).

[25] Lin R, Clowsley AH, Jayasinghe ID, Baddeley D, Soeller C (2017). Algorithmic corrections for localization microscopy with sCMOS cameras: Characterisation of a computationally efficient localization approach. Opt. Express.

[26] Araújo, Ó. T. *et al.* EMCCD in-situ periodic characterization in Shack-Hartmann wavefront sensor for GTCAO. In *Adaptive Optics Systems VI*, vol. 10703, 107034W, (International Society for Optics and Photonics, 2018). 10.1117/12.2312641.

[27] Hirsch M, Wareham RJ, Martin-Fernandez ML, Hobson MP, Rolfe DJ (2013). A stochastic model for electron multiplication charge-coupled devices-from theory to practice. PLoS ONE.

[28] Mortensen KI, Churchman LS, Spudich JA, Flyvbjerg H (2010). Optimized localization analysis for single-molecule tracking and super-resolution microscopy. Nat. Methods.

[29] Korevaar MAN, Goorden MC, Heemskerk JWT, Beekman FJ (2011). Maximum-likelihood scintillation detection for EM-CCD based gamma cameras. Phys. Med. Biol..

[30] Mortensen KI, Sung J, Flyvbjerg H, Spudich JA (2015). Optimized measurements of separations and angles between intra-molecular fluorescent markers. Nat. Commun..

[31] Mortensen KI, Flyvbjerg H (2016). “Calibration-on-the-spot”: How to calibrate an EMCCD camera from its images. Sci. Rep..

[32] Hynecek J, Nishiwaki T (2003). Excess noise and other important characteristics of low light level imaging using charge multiplying CCDs. IEEE Trans. Electron Dev..

[33] Robbins MS, Hadwen BJ (2003). The noise performance of electron multiplying charge-coupled devices. IEEE Trans. Electron Dev..

[34] English, B. P. & Singer, R. H. A three-camera imaging microscope for high-speed single-molecule tracking and super-resolution imaging in living cells. in *Biosensing and Nanomedicine VIII*, vol. 9550, 955008, (International Society for Optics and Photonics, 2015). 10.1117/12.2190246. 10.1117/12.2190246 PMC4724806 26819489

[35] Morisaki T (2016). Real-time quantification of single RNA translation dynamics in living cells. Science.

[36] Ryan DP (2020). A framework for quantitative analysis of spectral data in two channels. Appl. Phys. Lett..

