# Supplementary material for: A gain series method for accurate EMCCD calibration
Source: Sci Rep. 2021 Sep 15;11:18348. doi: 10.1038/s41598-021-97759-6 (PMC8443689; doi:10.1038/s41598-021-97759-6)
Supplement: Supplementary file 1 — Supplementary Information. [file 41598_2021_97759_MOESM1_ESM.pdf]

Supplementary information for: A gain-series method for accurate EMCCD calibration

Duncan P. Ryan[1,*], **Megan K. Dunlap**[2], **Martin P. Gelfand**[3], **James H. Werner**[1], **Alan Van Orden**[2], **and Peter M. Goodwin**[1]

[1]Center for Integrated Nanotechnologies, Los Alamos National Laboratory, Los Alamos, 87545, USA
[2]Department of Chemistry, Colorado State University, Fort Collins, CO 80523, USA
[3]Department of Physics, Colorado State University, Fort Collins, CO 80523, USA
[*]ryand@lanl.gov

S1 EMCCD Device Architecture

The general EMCCD device architecture involves multiple registers that each contribute specific noise characteristics. Figure S1 shows a simplified diagram of the registers. Photons are detected in the sensor pixel array. The emission and detection mechanisms follow Poisson statistics and are characterized by an average detection rate denoted E in the formalism of this work. The intensities are unique to the individual pixels in the detection array. In some modes of operation, the sensor array is copied into a storage array before readout. This secondary array does not change the production of photoelectrons. During readout, each row of the sensor array is moved into a readout register. From this point forward, all electrons from individual pixels are processed through the same registers, and the response parameters should be considered as global (some exceptions, as described in the manuscript and below). As a row of pixels is serially shifted though the multiplication register, the number of photoelectrons is amplified according to the average multiplication factor g. This process increases the noise of the signals at the output S. The final readout steps involve converting the voltages into count numbers with a ratio ϕ that introduces additional noise according to a normal distribution with width σ, and adding an offset S_0 that is independent of the signal levels.

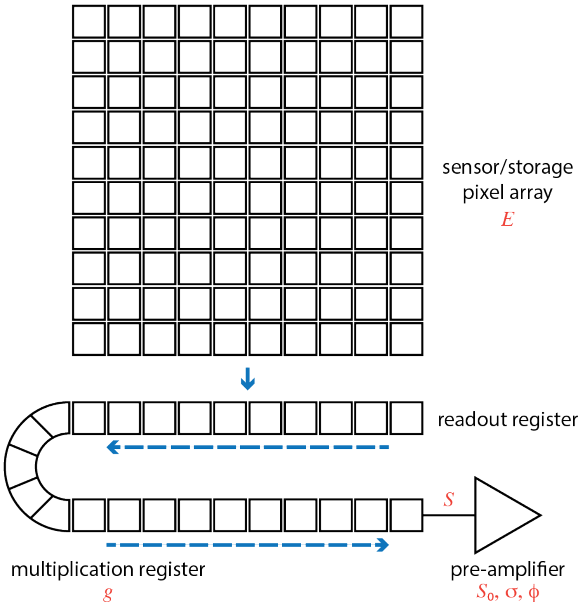

Figure S1. EMCCD device architecture. The components of an EMCCD camera involved with readout are: the sensor, the readout register, the multiplication register, and the pre-amplifier that converts electrons into digital counts. Red text indicates which registers are responsible the various components of EMCCD noise model. Variations to this general architecture exists, such as the dual readout registers that were a feature of the second EMCCD camera reported in this work (see Section S11).

S2 Noise Model Implementation in PyMC3

We used the statistical modeling package PyMC3 to explore the various calibration methods using maximum *a posteriori* probability (MAP) estimation and Markov chain Monte Carlo (MCMC) sampling. The PyMC3 package offers hardware accelerated symbolic math operations as well as a stable implementation of the No-U-Turn Sampler (NUTS). However, the native support for complex numbers, necessary for the readout noise convolution step using Fourier transforms, in the Theano dependancy is limited, requiring ad hoc implementations that ultimately increase computation time. Instead, we implemented the PGN camera noise model in TensorFlow with a Theano wrapper. Furthermore, future PyMC development will move away from Theano dependencies, supplanting them with TensorFlow for long term support. Finally, the move to an eager execution mode with the TensorFlow 2 release was not optimal for this implementation of the camera noise model, incurring a significant speed reduction. Therefore, the noise model was implemented with the TensorFlow 1 back-compatibility API. We provide as supplemental software the Python scripts to run the various calibration methods.

Two implementation changes were made to the explicit form of the PGN noise model in Eqn. (1). The PG noise model was implemented with the transformation

$$I_1(2\sqrt{ES\phi/g}) \quad \longrightarrow \quad \exp\left(2\sqrt{\frac{ES\phi}{g}}\right)\left[\exp\left(-2\sqrt{\frac{ES\phi}{g}}\right) I_1(2\sqrt{ES\phi/g})\right], \tag{1}$$

where the term in brackets is the exponentially-scaled modified Bessel function of order 1. This term can be substituted with the corresponding special function for which there are numerical methods. Using this functional form avoids floating-point errors that arise when the argument of the Bessel function is large—a potential occurrence given realistic parameter ranges.

Because non-integer ADU conversion factors ϕ result in scaled count units representing non-integer electron counts to which the discrete Poisson distribution does not apply, the non-multiplying noise model used for $1\times$ datasets was implemented with the continuous Gamma function

$$\ln q(S|E,\phi) = S\phi \ln E - E - \ln\Gamma(S\phi + 1) + \ln\phi. \tag{2}$$

Again, the use of the log-gamma special function avoids floating-point errors for potential large-valued arguments. The noise model for $1\times$ datasets is the convolution of the readout noise with the function $q(S) = \exp[\ln q(S)]$ using Eqn. (2).

The Bayesian hierarchical model allows individual datasets in a gain series to have separate photoelectron intensity levels $E^{(i)}$, but draws those random variables from a shared prior distribution. This model accommodates illumination intensity drift for measurements that are supposed to be connected together with the assumption of a shared illumination intensity. In this formulation, $E^{(i)} \sim \mathcal{N}(\overline{E}, \sigma_{\overline{E}})$ where $\overline{E}$ is the hyperparameter for the mean photoelectron intensity and $\sigma_{\overline{E}}$ is the hyperparameter representing the magnitude of the intensity drift. To avoid the inefficient sampling in hierarchical models (funneling) described by Wiecki[1], we implemented a non-centered approach for parameter sampling

$$\begin{aligned}
\overline{E} &\sim \mathcal{N}(\langle E_{\text{est}}\rangle, \sigma_a) \\
\sigma_{\overline{E}} &\sim \mathcal{H}(\sigma_b) \\
\mu^{(i)} &\sim \mathcal{N}(0,1) \\
E^{(i)} &= \overline{E} + \mu^{(i)}\sigma_{\overline{E}},
\end{aligned} \tag{3}$$

where $\langle E_{\text{est}}\rangle$ is the estimated intensity level based on all sample data and $\sigma_{a,b}$ are scaling factors of the normal ($\mathcal{N}$) and half-normal ($\mathcal{H}$) distributions. The model is largely insensitive to these scaling factors if they are selected within an appropriate order of magnitude. For this work, $\sigma_a = \sigma_b = 0.1\langle E_{\text{est}}\rangle$.

S3 Excess Noise Factor

The excess noise factor F^2 is a measure of the increased variance due to the electron-multiplying register in an EMCCD. The excess noise factor for impact-ionization devices—*e.g.* multi-channel plates (MCPs), electron-multiplying complementary metal-oxide-semiconductor sensors (EM-CMOS), avalanche photo-diodes (APDs), or EMCCDs—has been extensively studied[2,3]. When gain is low, the excess noise factor is not the value 2 that results with moment analysis of the PG noise model, Eqn. (2a). The works of Robbins and Hadwen[4], and Hynecek and Nishiwaki[5] derive formulations for the excess noise using a Bernoulli process for impact ionization. Figure S2 shows the dependence of the excess noise factor on the multiplication gain for these two models and the deviation at low multiplication. Sampling from the PG noise model (before readout noise is added) is also plotted to demonstrate how the noise model utilized in all calibration methods explored in this work does not capture the correct behavior at low gains. The PG noise model tends to a value of 2 for the excess noise, but shows inconsistency for small

photoelectron numbers. Despite the simplicity of the formulation, the PGN noise model does not accurately reflect the behavior of impact ionization devices, and results that rely on the PGN noise model may be inaccurate where the excess noise deviates from the factor of 2. Furthermore, recently proposed EM-CMOS devices show the excess noise factor is dependent on the gate architecture, potentially requiring a noise model tailored to the specific camera for accurate calibration[6].

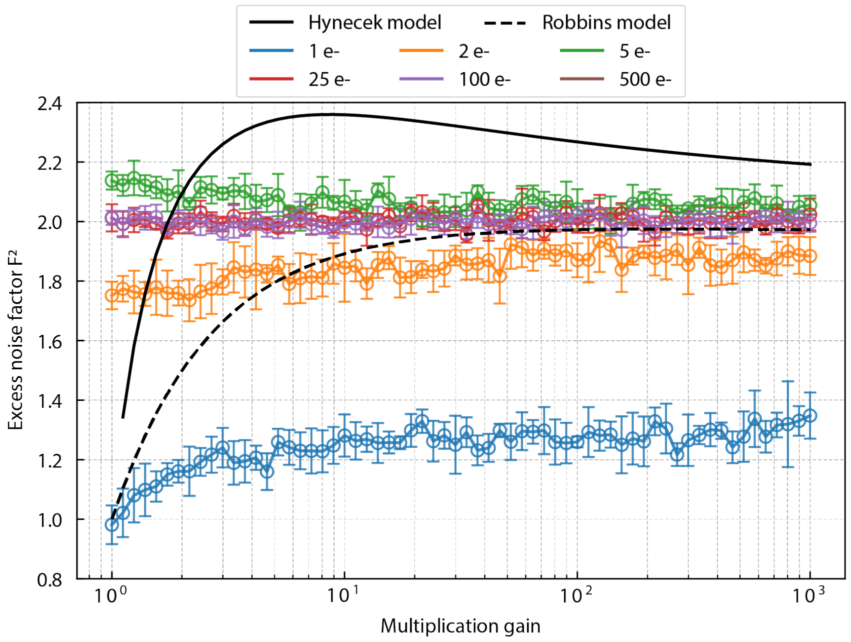

Figure S2. Excess noise factor. The Hynecek and Robbins excess noise models (512 amplification stages, black) deviate from the value of 2 for low gains. The distribution of the number of electrons after the amplification stage, given analytically by Eqn. (2a), was sampled and the excess noise factor of the noise model was calculated for several input photoelectron levels (color markers). The excess noise from the EMCCD noise model does not followed the behavior of the impact ionization process and is an approximation for the behavior of the amplification register.

S4 Information from Dark Images

The readout noise term in the EMCCD noise model also acts to offset the signal, which is necessary for camera readout to avoid negative ADU values. The offset occurs after the amplification stage and should be independent of the voltages applied to the amplification register. While this was mostly true for the 2009 camera, Fig. S3(b), we found this not to be the case for the 2012 ProEM camera, Fig. S3(a). The amplification stage can generate spurious charge (CIC noise) that is then amplified; however, this is not the source of the offset discrepancy. Spurious charge can be seen as tails to the distributions in Fig. S3 for the highest gain set-points. Unexpectedly, the offset value decreases as gain increases—a counter-intuitive behavior if the feature is due to the introduction of additional charges somewhere in the registers. We assume that this offset is due to an unknown process that does not affect the amplification or readout of true photoelectrons. Thus, the behavior can be treated by measuring offset maps for each gain set-point of a dataset.

S5 Pixel Uniformity

Pixel-wise calibration was done because (1) dark images indicated the offset parameter S_0 was not constant across an image, and (2) the illumination source produced an intensity gradient despite efforts to homogenize the illumination over the field of view. Figure S4 illustrates both of these difficulties for an ADU calibration dataset. The first panel is the dark image, averaged over 1000 frames. Regardless of the image acquisition size, readout artifacts were present, particularly in the edge columns and rows. Larger images were found to also exhibit the gradient, so cropping images could not generate uniform dark images. This behavior highlights the need to address offsets in the noise model directly, rather than with image processing and background subtraction. The middle four panels in Fig. S4 show the average images with illumination for an intensity series ADU calibration. With increasing intensity, the illumination eventually obscures the offset variations. However, there was also an illumination gradient. Thus, pixels cannot be grouped together in a single analysis because they do not share a uniform

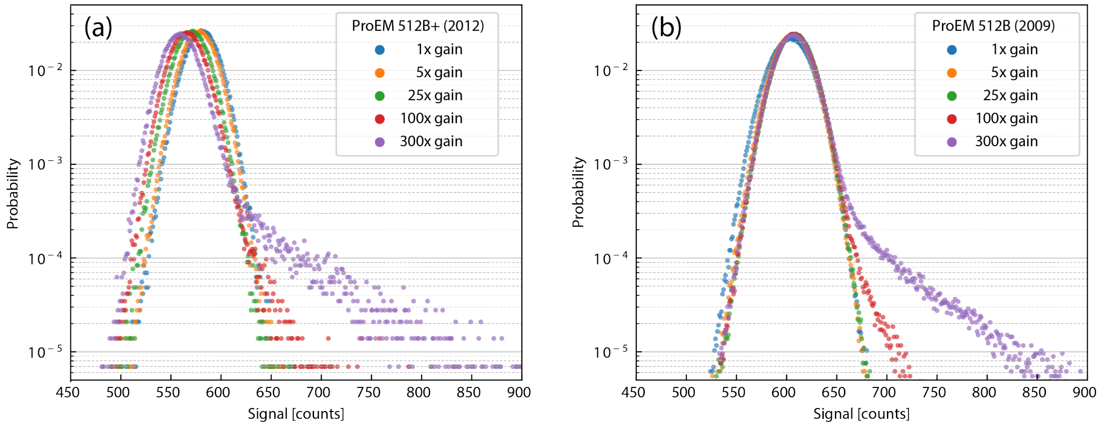

Figure S3. **Dark images.** Histograms from dark image sequences at various gain values for the **(a)** 2012 ProEM 512B+ and **(b)** 2009 ProEM 512B cameras. Data from a single pixel of each camera is shown. The peak is the normally distributed readout noise in the PGN noise model and the tails are from amplification of spurious charges generated in the multiplying register.

illumination intensity. In the final panel, results of the ADU calibration are depicted. Calibration using pixel-wise analysis generates a uniform map of ADU values. The offset and illumination intensity gradient features are not reflected in the final calibration when correctly accommodated in the analysis.

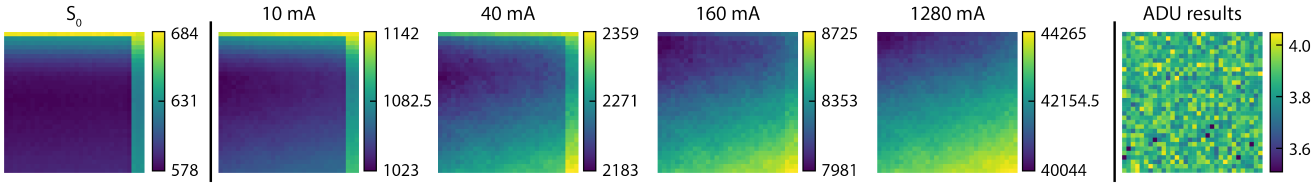

Figure S4. **Averaged raw frames from intensity series.** The first panel shows the dark image used to extract offset values S_0 for calibrating the ADU factor. Middle panels show the illumination intensity gradient for several intensity levels in the series. Panel titles indicate the LED source drive current (power level). The final panel shows the uniformity of the ADU calibration that resulted from this IS dataset.

S6 Stationarity of Calibration Data

Intensity-series calibration measurements require the illumination intensity to be constant over the duration of each intensity level. Gain-series calibration measurements are more stringent, requiring the illumination intensity to be constant over the duration of all calibration datasets. While a cursory examination of the time-series measurements does not indicate any drift of the mean or variance of the calibration signals, Fig. S5(a), we further applied a series of more rigorous statistical tests to demonstrate the constant illumination intensity of the LED source used for calibration measurements. A representative example of the autocorrelation function from a single pixel is shown in Fig. S5(b). The autocorrelation function immediately decays to zero, indicating no long term intensity drift.

Figure S5(c) shows the application of two statistical tests for stationarity to all data used for gain-series calibrations. The Augmented Dickey-Fuller (ADF) test and the Kwiatkowski–Phillips–Schmidt–Shin (KPSS) test report p-values for hypothesis testing. ADF tests against the null-hypothesis that the data is not stationary, while KPSS tests against the null-hypothesis that the data is mean-stationary. Thus, stationary data will have a small p-value for ADF and a large p-value for KPSS. All single-pixel time-series measurements of the calibration datasets used in this work passed the ADF test for stationarity. A very small portion of the data failed the KPSS test for stationarity. For those data that passed ADF but failed KPSS, this indicates the data was trend-stationary. However, for the purposes of calibration requirements, such strict stationarity is not necessary, and we can conclude the LED source was constant.

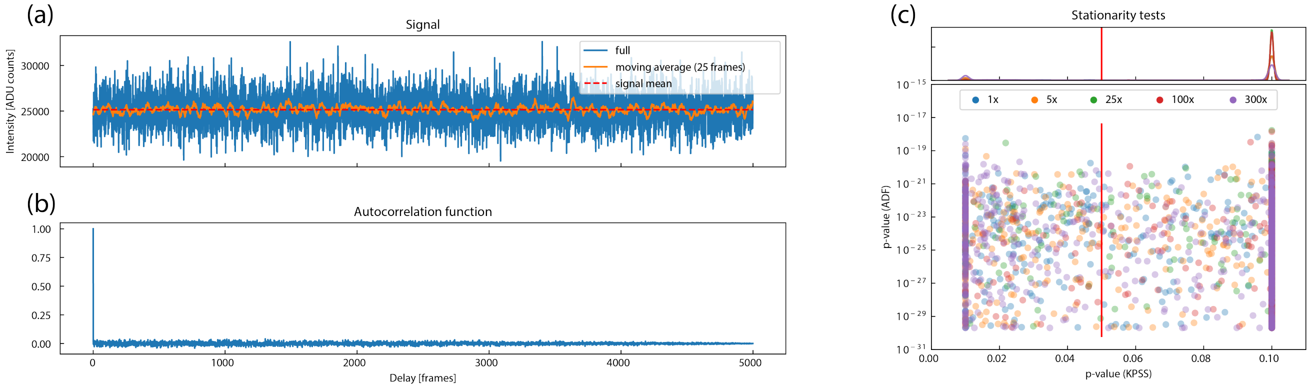

Figure S5. Stationarity tests. (a) The time trace of a single pixel from one measurement in a gain series. No obvious drift occurs over the 5,000 frames (250 s) of the measurement. **(b)** Autocorrelation of the same single-pixel data shows the correlation function decays immediately to zero, another indication that intensity drift does not occur and that the illumination source was constant. **(c)** Results from applying the ADF and KPSS tests to all calibration data from a gain-series calibration measurement (each pixel individually, all gain set-points). All pixels pass the ADF stationarity test, but a small population (histograms above scatter plot) fail the KPSS test.

S7 Covariance in the Noise Model

The degree of covariance between gain parameters (or ADU conversion factor) and photoelectron parameters is a characteristic of the EMCCD noise model. For example, a smaller gain value can be compensated for with larger photoelectron values and the likelihood value of either combination may change little. To illustrate this, Fig. S6(a) plots the distribution functions with the parameters determined by all the gain-series approaches (MLE with and without a 1× dataset and the hierarchical model) with the histograms of the calibration data. Within the probability range where the frequency of the data were observed, the three solutions are indistinguishable, despite estimating different and non-overlapping gain distributions. MCMC inference further demonstrates this point. Figure S6(b) shows the correlation from MCMC sampling between the 25× gain parameter and the shared photoelectron parameter for a single pixel (MLE without the 1× dataset). There is a strong linear relationship among the parameters. Parameter covariance such as this shows how the EMCCD noise model can accommodate different gain values given the calibration data. Thus, the various gain estimates given by each calibration method is due in part to the intrinsic flexibility of the EMCCD noise model.

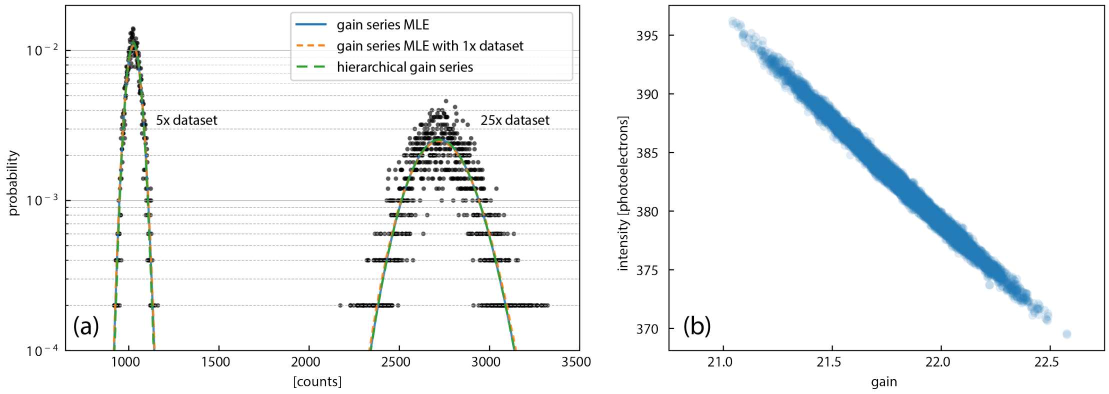

Figure S6. Correlation among fit parameters. (a) Fitting gain-series data to calibration models that share a single intensity level (blue), share a single intensity level including a no-gain dataset (orange), and using a hierarchical noise model (green) produce essentially identical distributions. However, each method produced parameters estimates with different combinations for the intensity and gain pairs, but the resulting distribution shapes are similar. Only the region including the 5× and 25× components are shown in the plot, but the other measurements in the gain-series were included for all analyses. **(b)** MCMC samples of the same pixel as (a) demonstrating the strong correlation between the 25× gain value and the intensity for the MLE method.

S8 Gain Linearity

The effective gains were approximately linear with gain set-point for the cameras tested in this work. Figure S7 shows the estimated gains as a function of their set-points. Datasets for intensity-series and gain-series methods were collected at only 1×, 5×, 25×, 100×, and 300× set-points. However, the linear response can be used to extract effective gains at other set-points in the case a specific calibration dataset was not recorded at the time of calibration.

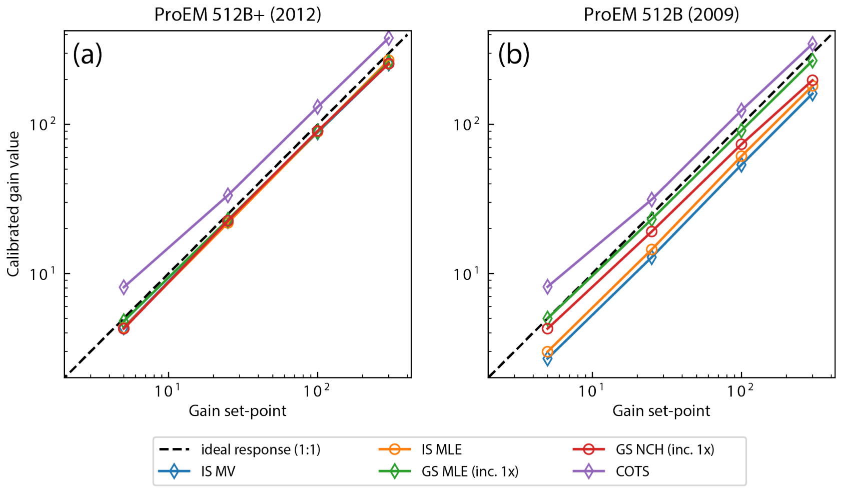

Figure S7. Gain linearity. The results from each calibration method for the **(a)** 2012 and **(b)** 2009 cameras as a function of the software set-point are shown. The gain response is approximately linear with the gain set-point for both cameras.

S9 ADU Conversion Factor Stability

While the gain calibration of a camera should be checked at regular intervals because the applied voltages in the amplification register can deteriorate, the ADU calibration is fixed. Figure S8 shows the results from two calibration datasets taken before and after a four month interval of frequent use. The ADU characterization was consistent and the calibration reproducible. Furthermore, there was no effort to match intensity levels between the two datasets, demonstrating the insensitivity of the calibration methods to exact illumination conditions.

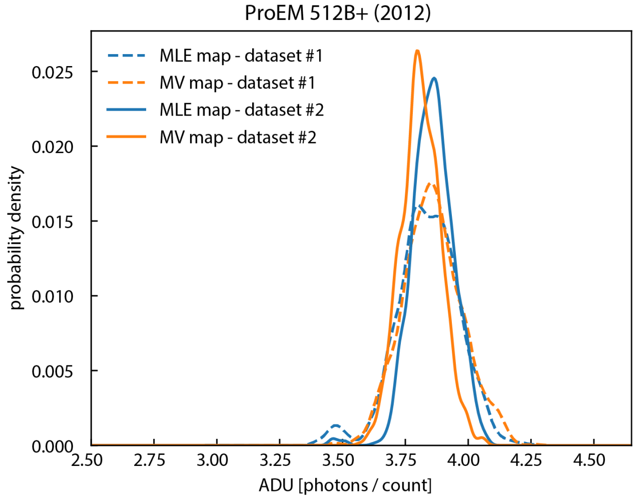

Figure S8. ADU calibration stability. Histograms of calibration results for ADU values before (dashed curves) and after (solid curves) a four month period of frequent use. Results from the traditional MV tests (orange) and the MLE method (blue) were consistent across the initial and recheck of the ADU calibration.

S10 Complete Gain Calibration Set

Figure S9 shows the complete gain calibration for the 2012 ProEM 512B+ camera, including the COTS gain estimates (supplemental figure to Fig. 3). The COTS results with their associated uncertainties for each of the ten bead measurements are shown in light purple, and the ensemble averages and the standard deviations of the bead ensembles are shown in dark purple. COTS results had greater uncertainty, and the results were always larger than the gain set-points. The IS and GS calibration methods always generated estimates smaller than the gain set-points.

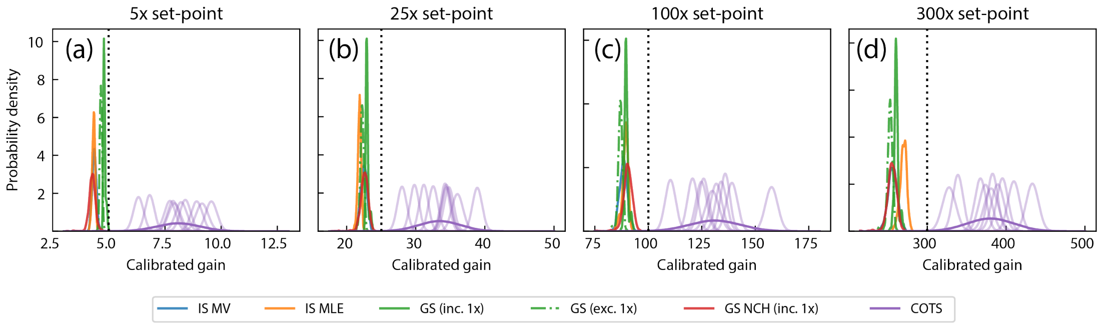

Figure S9. Full gain calibration results for 2012 ProEM 512B+ camera. A replot of Fig. 3 showing expanded axes to indicate the results of the COTS calibration method.

S11 Characterization for Second EMCCD

A second EMCCD camera was calibrated to test the various methods against a different device. Figure S10 shows the ADU calibration for a 2009 ProEM 512B camera. An unexpected feature of the ADU calibration was a column dependence: odd/even pixel columns had different ADU estimates (see insert and the solid/dashed distributions). This feature was apparent in both MV and MLE calibration methods. The device architecture of this older camera contains two readout registers with alternating columns of pixels passing through the different registers. This is not captured in the manufacturer calibration because the manufacturer's measurements were acquired on a single column of pixels. Furthermore, this camera exhibited more substantial differences among the manufacturer's, MV, and MLE calibration methods. In subsequent gain and localization analyses, column-dependent ADU values were paired with the matching pixel data.

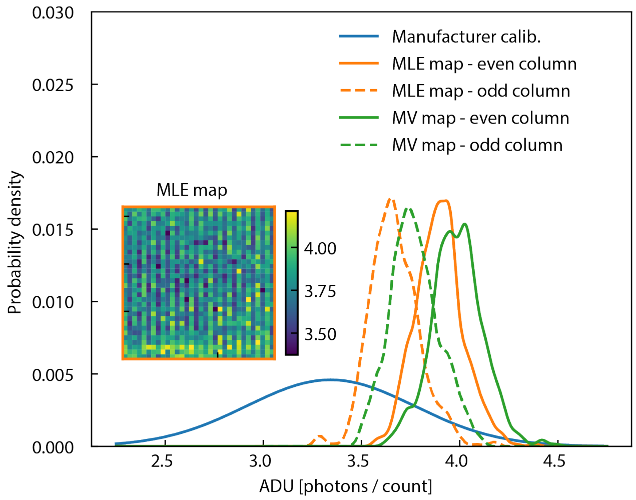

Figure S10. ADU calibration for 2009 ProEM 512B. Results from ADU calibration of an older ProEM camera. The dual-readout architecture is evident from the two distinct distributions present in both calibration methods. The insert illustrates the column-dependent artifacts introduced by the dual-readout architecture.

Results from IS, GS, and COTS gain calibration of the 2009 camera are shown in Fig. S11. Similar to the primary camera, IS

and GS calibrations estimated gain values lower than the gain set-points, while COTS consistently estimated larger values that showed greater uncertainties. IS and GS distributions were again determined from individually estimating the gain parameters from the 1024 pixels. Because the correct column-associated ADU values were used for gain calibration, the gain distributions are single-modal, demonstrating common gain values from the shared multiplication register despite the dual readout registers. The distributions for the older camera showed greater differences among gain estimates than the newer camera detailed in the manuscript.

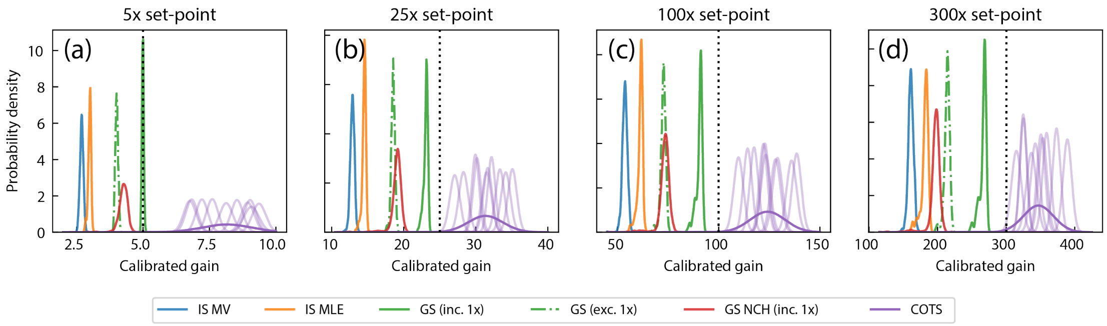

Figure S11. **Gain calibration results for 2009 ProEM 512B.** Complimentary result summary to Fig. 3 for the older ProEM camera. Gain estimates for the various series calibration methods were more distributed than the primary camera studied in this work.

Figure S12 shows the bead validation results for the older ProEM camera. The GS methods perform better than the IS, COTS, and NCH methods. Using GS results from a calibration set that included the $1\times$ data improved the consistency more than the newer camera demonstrated. The bead intensity results showed in Fig. S12 are from simultaneous measurements of the same beads as Fig. 5.

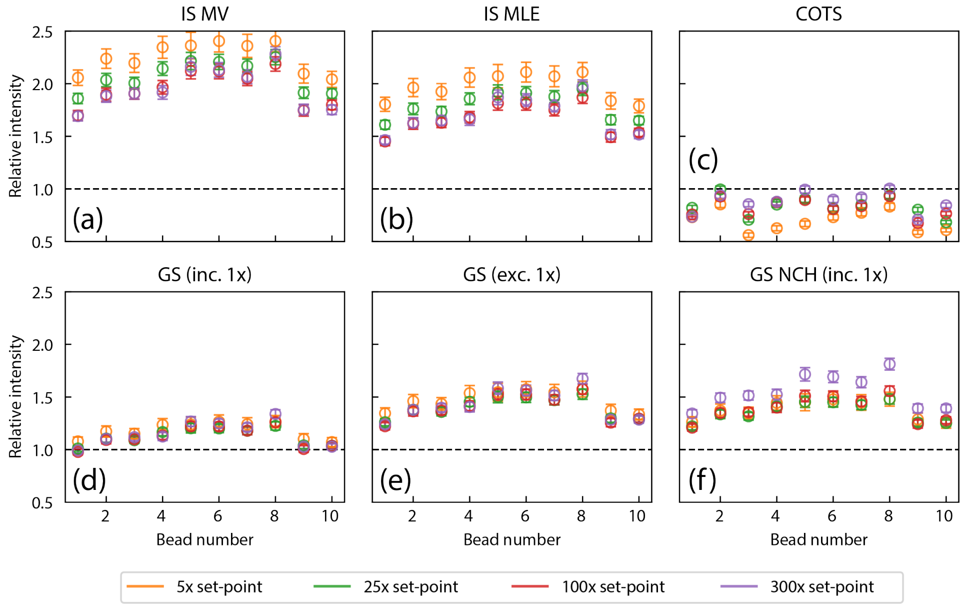

Figure S12. **Summary of bead results for 2009 ProEM 512B.** Complimentary results to Fig. 5 for the older ProEM camera. Similar trends were observed, with the gain-series methods outperforming other methods.

References

1. Thomas Wiecki. Why hierarchical models are awesome, tricky, and Bayesian. https://twiecki.io/blog/2017/02/08/bayesian-hierchical-non-centered/ (17).

2. van Vliet, K. & Rucker, L. Theory of carrier multiplication and noise in avalanche devices—Part I: One-carrier processes. *IEEE Transactions on Electron Devices* **26**, 746–751, DOI: 10.1109/T-ED.1979.19489 (1979).

3. Hollenhorst, J. A theory of multiplication noise. *IEEE Transactions on Electron Devices* **37**, 781–788, DOI: 10.1109/16.47786 (1990).

4. Robbins, M. S. & Hadwen, B. J. The noise performance of electron multiplying charge-coupled devices. *IEEE Transactions on Electron Devices* **50**, 1227–1232, DOI: 10.1109/TED.2003.813462 (2003).

5. Hynecek, J. & Nishiwaki, T. Excess noise and other important characteristics of low light level imaging using charge multiplying CCDs. *IEEE Transactions on Electron Devices* **50**, 239–245, DOI: 10.1109/TED.2002.806962 (2003).

6. Brugière, T., Mayer, F., Fereyre, P., Dominjon, A. & Barbier, R. A Theory of Multiplication Noise for Electron Multiplying CMOS Image Sensors. *IEEE Transactions on Electron Devices* **61**, 2412–2418, DOI: 10.1109/TED.2014.2320966 (2014).
